# Efficient Source Camera Identification with Diversity-Enhanced Patch Selection and Deep Residual Prediction

**DOI:** 10.3390/s21144701

**Published:** 2021-07-09

**Authors:** Yunxia Liu, Zeyu Zou, Yang Yang, Ngai-Fong Bonnie Law, Anil Anthony Bharath

**Affiliations:** 1 Center for Optics Research and Engineering (CORE), Shandong University, Qingdao 266237, China; eyxliu@sdu.edu.cn; 2 Shandong Key Laboratory of Storage and Transportation Technology of Agricultural Products, Shandong Institute of Commerce and Technology, Jinan 250103, China; 3 National Engineering Research Center for Agricultural Products Logistics, Jinan 250103, China; 4 School of Information Science and Engineering, Shandong University, Qingdao 266237, China; 5 Department of Electronic and Information Engineering, The Hong Kong Polytechnic University, Hong Kong 999077, China; ennflaw@polyu.edu.hk; 6 Department of Biomedical Engineering, Imperial College London, London SW7 2AZ, UK; a.bharath@imperial.ac.uk

**Keywords:** imaging sensors, source camera identification, convolutional neural network, deep learning, image forensics

## Abstract

Source camera identification has long been a hot topic in the field of image forensics. Besides conventional feature engineering algorithms developed based on studying the traces left upon shooting, several deep-learning-based methods have also emerged recently. However, identification performance is susceptible to image content and is far from satisfactory for small image patches in real demanding applications. In this paper, an efficient patch-level source camera identification method is proposed based on a convolutional neural network. First, in order to obtain improved robustness with reduced training cost, representative patches are selected according to multiple criteria for enhanced diversity in training data. Second, a fine-grained multiscale deep residual prediction module is proposed to reduce the impact of scene content. Finally, a modified VGG network is proposed for source camera identification at brand, model, and instance levels. A more critical patch-level evaluation protocol is also proposed for fair performance comparison. Abundant experimental results show that the proposed method achieves better results as compared with the state-of-the-art algorithms.

## 1. Introduction

Image content has become an important component of social media, driven by low-cost and ubiquitous image acquisition and network technology. In parallel, there are many image processing tools, providing powerful manipulations of the image. Images can be easily edited to cover up information for illegal purposes, and it can be difficult to distinguish edits with the naked eye. Therefore, tools for image forensics are in urgent need to verify the provenance and authenticity of images [1,2,3].

Source camera identification (SCI) is one of the topics that has received continuous attention in the image forensic community. The purpose of SCI is to determine the particular source camera used to shoot the digital image under investigation. Depending on the specific identification task, there is source camera identification at the instance level [4,5] (to determine the specific camera device), the model level [6,7,8] (to determine the camera model), and the brand level [9,10] (to determine the camera brand). By analyzing traces left by internal operations of the camera, SCI can be achieved independently of (such as the EXIF tag or JPEG header [11]), which is easily removed. Meanwhile, in contrast to watermarking techniques, which need to artificially add information to the original image, SCI has a wider range of applications, being a passive method. Source camera identification can assist in determining the owners of illegal and controversial materials, as well as helping to resolve the issue of image copyright, to a certain extent [7]. Patch-level SCI techniques can also be used to detect image forgery.

There have been many successful conventional methods for source camera identification. The rationale for robust instance-level SCI is that captured images are affected by certain imaging characteristics unique to the device, such as lens aberrations [11], sensor pattern noise (SPN) [12,13,14], white balance [15], and JPEG compression [16] parameters, etc. In the case of camera model identification, different built-in image processing algorithms and parameter settings, (such as color filter array (CFA) interpolation artifacts [17], JPEG quantization [18], demosaicing traces [19], DCT coefficients [20], etc.) adopted by different camera models may leave unobservable clues on captured images. Meanwhile, differences between camera manufacturers will leave weak traces in resulting images, which provides the foundation for brand-level source camera identification. To sum up, traces left by different camera instances, models, and brands are stable and irreversible. As features arising from the source camera are relatively weak in comparison to the perceptual image content, denoising operations are often utilized as the first step to extract residual images, guided by prior knowledge of relevant features, to achieve source camera identification. However, identification performance is greatly influenced by the imperfection of different denoising algorithms as accurate residual images are hard to estimate.

Driven by the rapid development of deep learning technology, a large number of deep methods have been proposed. Bondi et al. pioneered the first attempt [7] of camera model identification with convolutional neural networks (CNN). Many successful deep network structures in computer vision communication have been directly applied in the camera identification field—for instance, the CNN [8,11,21], ResNet [9], InceptionNet [22], DenseNet [23,24], and MobileNet [25]. In some cases, deep networks are utilized for feature extraction only, whereas camera identification is performed by other classifiers [21,25]. Moreover, there have also been networks designed specifically for source camera identification, such as the richer convolutional feature network-based representation [26], RemNet [27], and Siamese network-based works [28,29]. Other than the above works on the fixed data set, Sameer et al. studied the problem of blind identification of social networks images [30], whereas the open-set problem is discussed in [31,32] with shallow networks. Furthermore, the fast advent of sensor technology and proprietary in-camera processing algorithms equipped with modern smart sensors have imposed increasing challenges to the community [33,34]. There have been some recent studies on SCI methods that are robust to adversarial attacks [35,36]. Although promising results have been reported with the increase of accumulated data, network complexity and training costs have increased dramatically. These are obstacles to performance generalization and efficient implementation in real applications.

It is worth noting that preprocessing is of vital importance, where weak camera-related information is enhanced to be less influenced by image contents [8]. Popular choices of preprocessing modules are high-pass filter [8], normal convolutional layer [9], and constrained convolutional layer [28,31,37,38]. Considering that strong edges are mostly related to image content, the concept of selective preprocessing is proposed in [39] by Gaussian smoothing of strong edge patches. Meanwhile, data augmentation is another effective preprocessing method that usually leads to improved robustness [23,24]. With empirical mode decomposition (EMD) augmented data, the DenseNet method [24] won the first prize in camera model identification competition of IEEE Signal Processing Cup 2018. Combining nonlinear median filtered residuals, augmented convolutional feature maps proposed in [38] reported robustness against resampling and recompression. There are fully end-to-end methods that report better preprocessing performance. Remnant blocks are designed in [27], whereas an automatic residual extraction module is presented in our previous work [40].

Among all strategies for robust camera identification, patch selection deserves special attention for its simplicity and effectiveness. Only representative patches are selected for training; thus, computation complexity and possibility of overfitting are greatly reduced as compared to methods that utilize all patches for training [9]. It was revealed by experimental results in [10] that, without patch selection or preprocessing, the CNN-based approach is not as efficient as the SPN-based technique. The patch selection criterion based on local mean and variance proposed in [7] is followed by many works [22,24,27,32], whereas a similar strategy is proposed in [9] to train three parallel residual networks for different types of patches. Some others select central patches [11,29,31,38] or randomly select patches [10,23,28].

Since source camera identification methods have not been under development for a long time, a fair evaluation standard has not yet been formed. First, the scale, characteristics, regularity of image capturing process, rationality of training, and validation and testing sets division of experimental data sets are inconsistent. It is an essential prerequisite for the success of all data-driven-based learning methods. Second, performance evaluations are carried out either on whole image level [10] or by majority voting of several representative patches [7,22,27,32], or else on the individual patch level [11]. They are in increasingly difficult order, which makes direct identification-rate-based comparison unfair. Third, different methods are trained and tested on varying patch sizes (from 36×36, 64×64, 227×227, 256×256 to 512×512). Generally speaking, the smaller the image patch, the less camera information is involved, and the more difficult to achieve robust identification.

**Figure 1:**
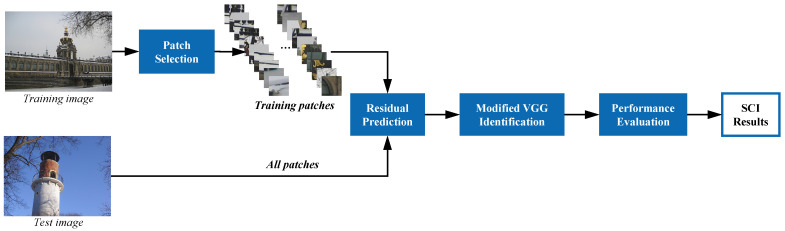
Framework of the proposed source camera identification method.

In this work, a patch level compact deep network for efficient source camera identification is proposed. Our explicit goal is to improve the effectiveness of source camera identification at all instances, models, and brand levels with controlled computing power. To this end, we follow a data-driven approach and exploit the patch selection and residual prediction design. Figure 1 illustrates the framework of the proposed method. In the training stage, only a small number of representative patches are selected as training data, where improved efficiency is obtained. This also improves the robustness and generalization ability of the deep network such that only intrinsic source camera-related features are learned. Furthermore, a specialized residual prediction module is designed to reduce the impact of image content on source camera identification. Finally, a modified VGG [41] network is utilized for subsequent feature extraction and classification. In the testing stage, all patches in testing images are identified according to the proposed performance evaluation protocol. The main contributions of this article are as follows:

- We propose a patch selection strategy based on local textural and semantic criteria, which are implemented by patchwise mean and variance scoring and *K*-means clustering, respectively. Training cost can be greatly reduced with enhanced diversity of the training data, thus, in turn, forcing the network to learn more intrinsic camera-related features for robust identification.
- A residual prediction module that automatically estimates residual image based on Res2Net [42] is proposed to reduce the impact of image contents. More granular multiscale richer features could be learned in a fully end-to-end manner, bypassing the drawbacks of traditional denoising methods due to imperfect filtering.
- Based on careful examination of the images in the Dresden database [43], we suggest a patch-level evaluation protocol for camera instance, model, and brand level experimental design method for fair comparison.

The organization of the paper is as follows. In Section 2, we review the related works of source camera identification. Details of the proposed source camera identification algorithm are discussed in Section 3 and Section 4, in which the evaluation protocol and experimental results are presented. Section 5 concludes the work.

## 2. Summary of Source Camera Identification Methods

### 2.1. Conventional vs. Deep Learning Methods

#### 2.1.1. Conventional Methods

Conventional methods rely on handcrafted features for source camera identification. Among all sensor-pattern-noise (SPN)-based methods, photoresponse nonuniformity noise (PRNU) [4] is the most accepted feature. Noise residual (*R*) is an important concept in PRNU estimation. It is obtained by subtracting a denoised version F(I) from the original image *I*:

(1) R=I−F(I),

where F(·) denotes certain filtering applied to *I*, either in form of a low-pass filter or an image denoising algorithm. In this way, image content is suppressed, and PRNU is then estimated accordingly. Identification is usually based on the statistical hypothesis test of normalized correlation coefficients. Further efforts are mainly focused on reducing the impact of image content [13], PRNU enhancement [44,45,46], and adoption of dual tree complex wavelet [47], with performance improvement reported.

There have been numerous model level features, including co-occurrence matrices [6,48,49,50], local binary patterns (LBP) [51,52,53], demosaicing features [19,48], generalized noise model [54], moments of 1D and 2D characteristic functions [55], heteroscedastic noise model [56] etc. In [57], it was proved that the SPN method is equally applicable to identification of camera models and camera brands. Moreover, combination of multiple features [49,51,58] is also a popular solution. Identification results are finally obtained by a machine learning classifier, where support vector machine (SVM) is the most popular choice. Methods proposed by [51,55,58] can also be applied to brand-level source camera identification.

A major appeal of all these conventional methods is their simplicity and interpretability as they are derived based on explicit or implicit models. However, they suffer from some drawbacks. First of all, accuracy is greatly influenced by varying image contents due to imperfect denoising algorithms. In addition, in-camera processing is certainly nonwhite. Consequently, performance degradation due to assumption deviation is unavoidable.

#### 2.1.2. Deep Learning Methods

Unlike the conventional feature engineering works guided by prior knowledge, deep learning methods follow a data-driven approach. Successful networks in computer vision society, such as AlexNet [8], ResNet [10,30], and DenseNet [23,24], are first applied to the field of source camera identification. With structure adjustment [8,9,10] or pretrained parameters [23,24,25], they perform well at the model and brand levels. Convolutional-neural-network-based shallow structures [7,31,39,59] are also prevalent in early years, where additional classifiers are sometimes cascaded after for better performance [7,21,32,38]. The importance of preprocessing layers is justified in [31,38,40], echoing the noise residual concept in SPN-based conventional methods. The recently proposed RemNet [27] method also exploits this property where a special remnant block is designed to dynamically suppress image content.

There are some efforts utilizing parallel networks [9,22,24] or multiple combined networks [24,28] for better performance. Three branches of ResNet are adopted for feature learning at different spatial scales in [9], whereas Inception-ResNet and Xception Network are adopted in parallel for feature extraction in [22]. DenseNet-201 and Squeeze-and-Excitation block are combined in [24], while the similarity network is combined with a specifically designed network for image comparison in [28]. Performance gain is usually obtained by fusion of richer features. Furthermore, there have been some recent attempts to design deeper and more complicated network structures [11,27,29], where performance improvement is reported at the expense of high training cost.

In summary, deep learning solutions are emerging as strong candidates for SCI. Considering the special characteristics of camera identification application, how to exploit prior knowledge obtained from conventional methods is a fruitful direction in deep networks design. We will discuss our effort in patch selection and residual prediction module design in Section 3.

### 2.2. Patch Selection Schemes

As discussed previously, patch selection is a simple yet effective method in source camera identification. The scheme proposed by Bondi et al. [7] based on edge and textual evaluation of local patches is widely accepted [22,24,27,32]. The input image *I* is first divided into *m* nonoverlapping 64 × 64 × 3 patches $I=\left\{I_{1},I_{2},\cdots ,I_{m}\right\}$, where boundary parts less than 64 × 64 are ignored. Guided by prior knowledge from conventional methods that patches with more textures, edges and the mean value close to half of the image dynamic are more distinctive in camera identification, and a score *f* is defined as:

(2) $$\begin{array}{ccc} f_{i}=\frac{1}{3}\sum_{c\in [R,G,B]}[\alpha \cdot \beta \cdot \left(\mu_{ci}-\mu_{ci}^{2}\right)+\left(1-\alpha \right)\cdot \left(1-e^{\gamma \sigma_{ci})}\right], & & i\in (1,2,\cdots ,m) \end{array}$$

where $\mu_{ci}$ and $\sigma_{ci}$ are the mean and standard deviation of the *R*, *G*, and *B* color channels (normalized into the range of [0,1]) of the *i*th patch, whereas α, β, and γ are constants set to be 0.7, 4, and ln(0.01) according to [7]. All patches are then ordered according to *f*, where the top *T* patches are selected for training.

A similar scheme is proposed by Yang et al. [9] in which, based on local mean and standard deviation, all patches are categorized into three subsets according to the difficulty of classification:

(3) $$\left\{\begin{array}{ccc} \mathrm{Saturated} & & \mu \in [0,5]\cup [250,255],\sigma \in [0,25]\\ \mathrm{Smooth} & & \mu \in [0,5]\cup [250,255],\sigma \in [25,50]|\\ & & \mu \in [5,250],\sigma \in [0,50]\\ \mathrm{Others} & & others \end{array}\right.$$

where threshold values are determined empirically. The first difference between the edge and textural scheme [7] is that all patches are utilized for training. Three parallel ResNets are further employed to deal with these three subsets, respectively. This divide and conquer strategy brings prominent performance improvement. However, training cost is increased dramatically.

There have been other patch selection schemes. For instance, center patches are selected in [11,31,38] or conducted randomly [10,23,28]. However, all of these patch selection schemes are based on a single criterion. Thus, data diversity, crucial to success of data-driven methods, is hard to guarantee.

### 2.3. Preprocessing Methods

A notable characteristic of camera identification is that distinctive features are weak as compared with scene content. Both conventional and deep learning methods heavily suffer from this drawback. To solve this problem, various preprocessing methods have been proposed, including plain convolutional layer [9], LBP [52], 2D empirical mode decomposition (EMD) [24], Laplace edge detection filter and Gaussian filter [39], augmented convolution feature maps [38], and noise pattern [59], etc.

The most popular category of methods are based on the noise residual concept in PRNU estimation. Imposing a fixed high-pass filter [6,8] or some image denoising algorithm [4,60] can reduce the influence of image scene in some extent. However, it is difficult to get rid of artifacts introduced by imperfect filtering, which is a main disadvantage of conventional methods.

Residual prediction is also an important module in deep-learning-based methods. In [10], sequential multiscale high-pass filters are adopted for residual image prediction:

(4) $$\begin{array}{cc} I & =I-F_{1}\left(I\right)+F_{1}\left(I\right)\\ \; & =N_{1}+F_{1}\left(I\right)-F_{2}\left(F_{1}\left(I\right)\right)+F_{2}\left(F_{1}\left(I\right)\right)\\ \; & =N_{1}+N_{2}+F_{2}\left(F_{1}\left(I\right)\right)-F_{3}\left(F_{2}\left(F_{1}\left(I\right)\right)\right)+F_{3}\left(F_{2}\left(F_{1}\left(I\right)\right)\right)\\ \; & =N_{1}+N_{2}+N_{3}+F_{3}\left(F_{2}\left(F_{1}\left(I\right)\right)\right) \end{array}$$

by successively subtracting the output results of these three Gaussian filters. This method also suffers from the risk of image characteristics change as three sequential high-pass filtering operations are applied.

The constrained convolutional layer [37] initially proposed for manipulation detection is well applied in source camera identification [28,31,38]. By restricting a high-pass filter of the convolutional kernel ω by:

(5) $$\left\{\begin{array}{c} \omega_{k}^{\left(1\right)}\left(0,0\right)=-1,\\ \sum_{m,n\neq =0}^{k}\omega_{k}^{\left(1\right)}\left(m,n\right)=1, \end{array}\right.$$

fully end-to-end manner residual prediction is achieved, where $\omega_{k}^{\left(1\right)}\left(m,n\right)$ denotes the *k*th filter coefficients in the first layer at corresponding position (m,n). However, only three constrained kernels are learned and applied to the green channel of input color images, which is insufficient for robust identification.

## 3. The Proposed Source Camera Identification Method

As illustrated by the framework of the proposed system in Figure 1, all training and test images are first divided into nonoverlapping patches, which are set to be 64×64 in this work. In the training phase, representative patches obtained by patch selection module serve as training data to supervise the learning of subsequent residual prediction and classification. Once the parameters are trained, all patches in test images are identified for final performance evaluation.

In the following subsections, we will discuss how to use patch selection for enhanced data diversity and describe the design of noise residual prediction and classification module. Details of the evaluation protocol are also provided.

### 3.1. Multiple Creteria Based Patch Selection

The importance of patch selection has been justified by many works [7,9], while improved efficiency and robustness are obtained as compared to training with all image patches [9]. However, most patch selection methods are performed according to single criterion, which may cause inconsistency between training and testing data. For instance, if only patches with sharp edge are selected for training, there is high risk that subsequent CNN would be enforced to learn interpolation features near edges rather than source-camera-related information. As a consequence, networks trained on these selected patches may not work well in testing phase where all patches are identified.

To this end, a patch selection method based on multiple criteria for enhanced diversity of selected patches is proposed. Our explicit goal is to select a small number of patches that is representative of the underlying distribution of all training and testing patches. Prior knowledge obtained from conventional methods is utilized to guide criterion design.

First, the edge and textual criterion [7] is adopted based on local mean and variance evaluation, given the fact that more interpolation-related information is contained in such patches. For each training image, the top *T* patches with highest scores by (1) are selected. In this way, high-quality edge and textual patches are included in training patches.

Second, considering the low signal-to-noise ratio (SNR) of source-camera-related information with respect to image intensity, the semantic content is adopted as the second criterion for patch selection. In order to achieve better perceptual quality, camera manufacturers adopt different built-in processing algorithms for varying image contents. It has been revealed that the fingerprints left by the same camera are not identical to each other for different contents [9] on multiple shoots of images. Consequently, it would be helpful if more patches with similar contents are selected for training. The conventional unsupervised *K*-means algorithm [61] is adopted to perform the semantic clustering, due to its simplicity and effectiveness. This contributes another *K* patches into the training set.

Furthermore, several techniques are utilized for effectiveness and implementation efficiency. First, instead of directly clustering all nonoverlapping patch candidates into *K* clusters, a technique in which all patches are clustered into *k* clusters, where the first *n* patches closest to the cluster centroids are selected (K=k×n), is utilized for better discrimination performance. In this way, the *n* patches in each cluster are similar with each other, which will benefit the discrimination of instance level identification. Second, directly clustering in the original pixel space (4096×3) could be computationally prohibitive. The proposed solution is to use the patchwise mean and standard deviation as a feature vector ζ=(μ,σ), that later clustering is performed in this two-dimensional feature space. The proposed patch selection algorithm (Algorithm 1) is summarized as:
**Algorithm 1:** Multiple-Criteria-based Patch Selection**Input:** Image patch set, $I=\{I_{1},I_{2},\cdots ,I_{m}\}$ Number of textual patches, *T* Number of cluster centers, *k* Number of patches per cluster, *n* Number of iterations, *N* // *Edge-and-Texture-based patch selection* **for** i=1,2,…,m **do**  Calculate $f_{i}$ according to (2) **end for** Sort $f_{i}$ in descending order $f_{\pi (1)},f_{\pi (2)},\cdots ,f_{\pi (m)}$ Select the first *T* patches as edge and textual representatives: $E=\{I_{\pi (1)},I_{\pi (2)},\cdots ,I_{\pi (T)}\})$ // *Semantic-content-based patch selection* **for** i=1,2,…,m **do**  
$\mu_{i}=\frac{1}{3}\sum_{c\in [R,G,B]}\mu_{ci}$  
$\sigma_{i}=\frac{1}{3}\sum_{c\in [R,G,B]}\sigma_{ci}$ **end for** Form feature space $Z=\{\zeta_{1},\zeta_{2},...,\zeta_{m}\})$ from patch set $\zeta_{i}=\left(\mu_{i},\sigma_{i}\right)$ Perform *K*-Means clustering in feature space Z to obtain the *k* Cluster centroids: $c_{1},c_{2},\cdots ,c_{k}$ until *N* iterations is exceeded For each of the *k* centroids, select *n* nearest patches as semantic representatives: $S=\{I_{c_{11}},I_{c_{12}},\cdots ,I_{c_{1n}},\cdots \cdots ,I_{c_{k1}},I_{c_{k2}},\cdots ,I_{c_{kn}}\}$  **Output:** Training patch set P=E∪S

There is the possibility that some patches may be simultaneously selected by multiple criteria. A feasible solution under such circumstances is to preferentially retain patches by the semantic content criterion, so that the next ranked *c* patches according to the edge and textual score $\left\{I_{\pi (T+1)},I_{\pi (T+2)},\cdots ,I_{\pi (T+c)}\}\right)$ are merged into P, where $c=\left|E\cap S\right|$ is the number of commonly selected patches.

**Figure 2:**
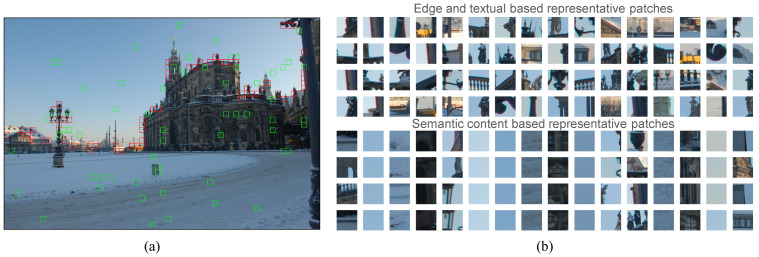
Illustration of multiple criteria-based patch selection. (**a**) Selected edge and textual patches (in red square) and semantic representatives (in green square); (**b**) visualization of selected patches.

A comparative example of the multiple-criteria-based patch selection is shown in Figure 2, where (T,k,n,K)=(64,16,4,64). Figure 2a visualizes the spatial position of selected training patches. It can be observed that edge-and-textual-based patches (highlighted in red squares) are mainly concentrated along the edge areas of the church and other buildings. Obviously, they are not typical enough to well represent the underlying input image. Network trained only on these patches cannot learn sufficient features to identify all patches during the testing phase. However, being representatives to report the majority of the whole scene, content-based selected patches (highlighted in green squares) cover varying contents covering the main scene of the image such as sky, ground, and the interior of the building. They serve as a good complement to the diversity of the training data, so that richer features could be learned from. Selected patches are shown in Figure 2b for further visual inspection, where the 16 columns corresponding to the centroids that we set for content-based patch selection. We see that the 4 selected patches are similar with each other, which will add to the diversity of selected patches for network training.

To summarize, the two textual and semantic content criteria adopted are orthogonal to each other, just as the color and shape attributes when identifying an apple. As a result, one can expect performance improvement in SCI, as diversity of selected training patches is well enhanced.

### 3.2. Residual Prediction Module

The identification result is vulnerable to image content if selected patches are directly fed into a CNN, despite the excellent feature learning capacity of CNNs. The importance of the residual prediction module has been verified by several works. An intuitive way for residual prediction would be ResNet [62] proposed by He et al., which has been successfully applied in SCI [9,10]. However, it is used for identification rather than learning residuals.

**Figure 3:**
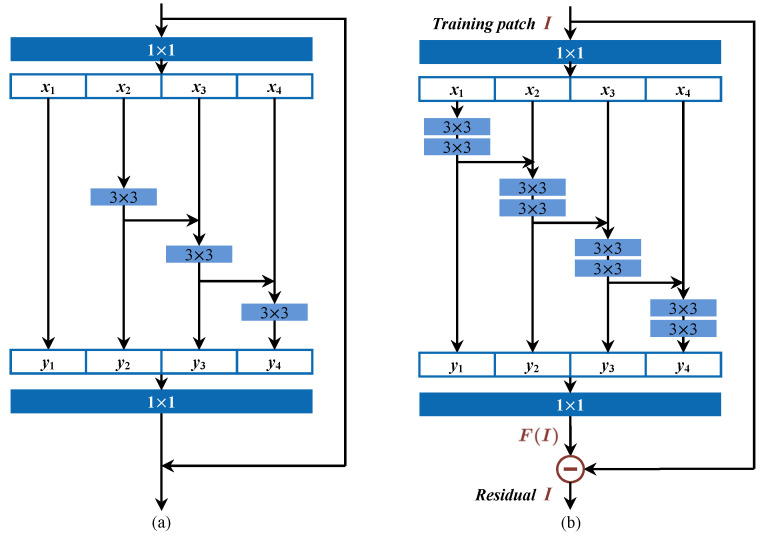
Network structures of the (**a**) Res2net. Reprinted with permission from ref. [42] Copyright 2019 IEEE and (**b**) the proposed residual prediction module.

Recently, a new multiscale backbone Res2Net [42] was proposed (shown in Figure 3a). By imposing hierarchical residual-like connections between smaller groups, it demonstrates consistent superiority in several tasks. Considering that local relationships are critical in SCI applications, more granular level multiscale properties should be further exploited in deep network design. Inspired by Res2Net, we propose a residual prediction module to reduce the impact of image content, which is depicted in Figure 3b. Several modifications have been made to explore richer features at a more granular level.

First, for each input training patch *I*, feature maps after 1×1 convolutional filters are evenly split into *s* groups, where *s* denotes the added scale dimension in addition to existing dimensions of depth, width, and cardinality (s=4 as depicted in Figure 3). The greater *s* is, the greater the number of granular level characteristics that can be learned by the network. As our emphasis is to exploit granular level features, two 3×3 convolutional layers are applied to each group, including the first group, which is directly passed to the output in Res2Net [42].

Second, subsequent group and output feature maps of the previous group are sent to the next sets of two 3×3 convolutional filters. This process is repeated several times until all feature map groups have been processed. By increasing one more 3×3 layer for each group as compared with [42], more equivalent feature scales could be obtained as the reception field sizes are enlarged whenever it passes a 3×3 filter. As the residual prediction module locates at the beginning of the deep network, and as more local relationships are exploited, better identification results can be expected.

Finally, feature maps from all groups are concatenated and fused together with another group of 1×1 filters. Hereby, we specially fix the output dimension to 3 to match the cardinality of input color channels. In this way, patch-to-patch residual learning can be achieved. An interesting outcome is that if we consider the learned features as F(I), by subtracting from the original patch *I*, we can obtain a residual image like *R* that has the same physical meaning comparable to its initial definition in conventional method as in (1). In other words, the deep residual prediction module could be considered as an adaptive denoising filter *F*. This provides us the possibility to bridge the gap between conventional and data-driven deep learning methods, which will facilitate better understanding and solving of the SCI problem.

**Figure 4:**
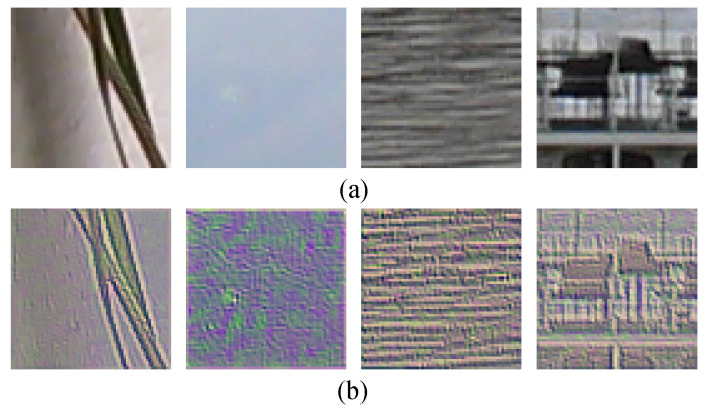
Visulization of typical pathes by the residual prediction module. (**a**) Original patches; (**b**) residual patches.

To illustrate the proposed residual prediction module, some example residual patches are shown in Figure 4. It can be observed from the comparison results that the influence of the varying image content has been reduced, whereas certain features are enhanced. For the first patch with strong edges, the residual prediction output lies consistently along the edges, whereas the fine-scale components are enhanced in the smooth region. This is more obvious for the second smooth patch where color interpolation-related features are supposed to be crucial for identification. With the proposed residual prediction module, granular level fine-scale local relationships are better exploited. Moreover, rich high-frequency details are retained for random or structural texture patches shown in the last two columns.

### 3.3. Modified VGG for Identification

After a fine-grained multiscale residual image has been obtained, a classification module is followed to further extract camera-source-related features. A modified VGG network is proposed in this work due to the simplicity and effectiveness of the backbone network.

The VGG network was initially proposed in [41] for classification and localization, and has been well generalized to various tasks and data sets. By discarding large convolutional kernels, the number of parameters has been greatly reduced as compared with early network structures. Meanwhile, given the residual image as input, VGG is more suitable for SCI applications as multiple consecutive 3×3 convolutional layers with pooling at different stages allows for better exploration of spatial relationships at varying scales, when compared with 1×1 kernels widely applied in deeper ResNet-based structures. Moreover, it is easier to train as a relatively shallow network.

**Figure 5:**
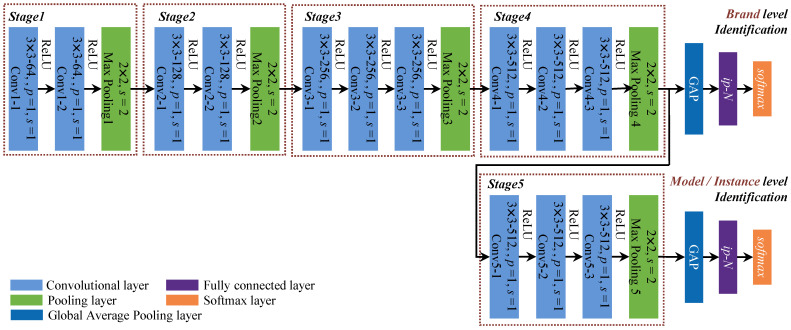
Framework of the proposed source camera identification method.

The proposed network shares similar structure with VGG, whereas specific parameters are shown in Figure 5 (*p* and *s* represent padding and stride parameters). For model and instance level SCI, more elaborate features should be learned. Although they share the same network architecture, training of instance-level network is based on fine-tuning of the pretrained model-level network. In contrast with this, common features lead to more stable brand level identification. Consequently, only the first four stages are involved in brand-level SCI.

Multiple consecutive 3×3 convolutional layers are divided into five stages, where ReLU activation and max pooling are connected afterward. In order to reduce the number of parameters, a global average pooling (GAP) layer is adopted in the proposed structure to replace the two fully connected layers for feature fusion. Finally, the attribution to the classification result is achieved by a fully connected layer (represented as ip*-N* in Figure 5) and the softmax function, where the number of neurons *N* is identical to the number of classes to be identified.

### 3.4. Performance Evaluation

Credibility of the experimental results has been greatly reduced as the experimental design of different SCI works is not uniform. In order to fairly compare the performance of SCI algorithms, a performance evaluation protocol is proposed with the following guidelines. It is based on the Dresden database [43], which is the largest and most widely accepted public image forensic database.

First of all, construction of the training, validation, and testing data sets is of vital importance to performance comparison fairness.

- For SCI task at one specific level, classes with only one instance at its lower level should be removed. For example, the “FujiFilm” brand is eliminated from brand level identification, as there is only one camera model “FujiFilm_FinePixJ50” in the Dresden data set. The possible influence of misleading the network to learn model level features could be avoided in this way. A similar principle applies to the model level SCI that models with only one instance are excluded. Instance-level SCI is not influenced such that all 74 camera instances are utilized.
- In order to reduce the effect of image content, scenes in the training set, validation set, and test set should be exclusive to each other. SCI algorithms are greatly affected by image content; images obtained from the same scene will affect the identification result severely. This is implemented with the scene number identifier of the Dresden database.

In the second place, a more critical patch-level evaluation method is proposed. Some identification is performed on the whole image [10], whereas some are based on majority voting of several patches [7,22,27,32]. In the proposed method, all 64×64 testing patches should be evaluated independently. Identification accuracy is calculated as:

(6) $$\begin{array}{c} Accuracy=\frac{\mathrm{No}.\mathrm{of}\;\mathrm{correctly}\;\mathrm{classified}\;\mathrm{patches}\;}{\mathrm{Total}\;\mathrm{No}.\;\mathrm{of}\;\mathrm{test}\;\mathrm{patches}}\times 100\%. \end{array}$$

It is more critical as a patch-level evaluation with no further strategy (such as voting) allowed. In this way, more valuable identification results could be obtained. The dyadic patch size of 64×64 is recommended, as it is more convenient to manipulate and adapt to different application scenarios, such as image manipulation detection, forgery detection, and so on.

## 4. Experiments

### 4.1. Experimental Step

Experiments were conducted to demonstrate the effectiveness of the proposed method. As shown in Figure 1, selected representative patches were utilized in the training and validation phases, while all patches in the testing images were identified in the testing phase.

In our experimental methodology, first, individual parts of the proposed algorithm, namely the patch selection scheme, residual prediction part, as well as the identification network, are compared while keeping the other parts the same. Camera model level results are reported at this stage, as it is the intermedium between brand- and instance-level identification, and is mostly studied in SCI applications. Then, the proposed algorithm is compared with state-of-the-art SCI methods on all brand, model, and instance levels with failure analysis. Application in image tampering detection is also presented.

For brand level identification, six camera brands were included, namely Agfa, Canon, Nikon, Pentax, Samsung, and Sony, which yields a training set with 6438 images. There are 1110 and 378 images in the validation and test sets, respectively. For model level identification, the similar models “Nikon_D70s” and “Nikon_D70” are merged into “Nikon_D70” as suggested in [7,27]. Detailed information of the 18 selected camera models is shown in Table 1, where the training set, validation set, and test set contains 7938, 1353, and 540 images. All camera instances in the Dresden database are adopted in instance level identification, with more than 10,000 images in the training set and 2193 and 2199 images in the validation and test sets, respectively.

Experiments were conducted on a PC with Intel (R) Core (TM) i5-8500 CPU @ 3.00 GHz, equipped with a NVIDIA GTX 1080Ti GPU on Ubuntu 16.04 operating system under the Caffe framework. The learning rate was initialized to 0.01 and the maximum number of iterations was 100,000. We set the weight decay to 0.00075 and the momentum to 0.9. The stochastic gradient descent (SGD) optimization algorithm was utilized, and the batch size was 64.

### 4.2. Experiment 1: Determination of Patch Selection Paremeters

To determine the parameter settings in the proposed patch selection algorithm, we carried out model-level experiments with the modified VGG identification network discussed in Section 3.3.

First, to determine the number of representative patches, we varied the number of selected patches for training from 32 to 256; the comparison results are shown in Table 2. Generally speaking, identification accuracy increases when more patches are involved in training, as more intrinsic features could be learned. However, the increase of training computation burden rises greatly, in sharp comparison with the slower growth in performance. In comprehension of the performance and computation cost, the number of representative patches was set to 128 according to the results in Table 2. The number of validation patches was also set to 128 per image due to consideration of computation cost.

Second, to determine the optimal parameter settings of the number of edge and textual patches *T*, number of cluster centers *k*, and number of patches per cluster in semantic content criterion *n*, we tried different combinations with the constraints that T+k×n=128. This resulted in 1,016,064 and 173,184 patches in the training and validation sets for model-level identification, respectively. Identification accuracies are shown in Table 3. The setting of (T,k,n)=(128,0,0) corresponds to the edge and textural scheme in [7] and serves as a baseline for comparison. From the comparison results, we can safely conclude that combining semantic content criterion indeed brings performance improvement due to enhanced diversity in training data. Among the varying combinations, we chose (T,k,n)=(64,16,4) as the final parameter setting, as it leads to the best performance.

**Figure 6:**
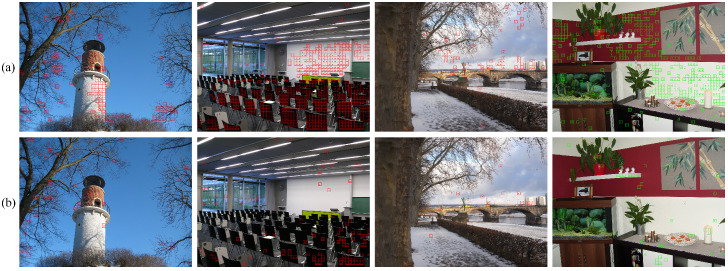
Visualization of misclassified patches with (**a**) patch selection scheme in [7] and (**b**) the proposed patch selection scheme.

Furthermore, to better understand the effectiveness of the proposed multiple criteria-based patch selection scheme, we compare with the patch selection scheme in [7] while keeping all other settings the same. Misclassified patches are depicted in Figure 6. The four images shown are captured by cameras from “Canon_Ixus70” (Model 0), “Panasonic_DMC-FZ50” (Model 8), “Ricoh_GX100” (Model 11), and “Samsung_NV15” (Model 14), respectively, where camera models are indexed by model number given in Table 1. Misclassified patches are highlighted with red (green in last image to distinguish from the large red background area) squares, where the number in the center indicates the incorrect camera model to which it has been assigned.

From the comparison, we see that the number of misclassified patches are greatly reduced with the proposed patch selection scheme. As revealed by Equation (2), the patch selection scheme in [7] put too much emphasis on edge and texture regions that patches in smooth regions are merely involved in training. This is the reason for the bad performance in the smooth regions of the red, white background and black back of the chairs in the two indoor images. In fact, there are rich source camera features in smooth regions, which are common in image content. Most patches in these areas are successfully identified (shown in Figure 6b). Similar improvement can also be observed in the tower, branches, and sky regions in the two outdoor images, benefiting from the enhanced data diversity with the proposed patch selection scheme.

### 4.3. Experiment 2: Comparison of Preprocessing Methods

To show the effectiveness of the proposed residual prediction module, we compared commonly used preprocessing methods on model level. For comparison fairness, all results were reported based on training the proposed modified VGG network with the multiple-criteria-based patch selection scheme. The results are summarized in Table 4.

There are cases in which no residual prediction is involved where selected patches are directly forwarded to the identification network [7,11,23]. The accuracy rate is only 87.37%, indicating the importance of residual prediction in forensic applications. Meanwhile, it is common to utilize a traditional filter [38,52,59] to smooth the image and residual is obtained by Equation (1). The 3×3 mean filter is a simple yet effective choice, which is implemented with the “cv2.blur” function of the OpenCV library [63] in our simulation. An interesting observation is that it slightly outperforms the fixed high-pass filter method [8].

The constrained convolutional layer method [38] could be trained in conjunction with the identification network; thus, it is more efficient as a fully end-to-end feature method. We set the kernel size to be 5×5 as in their original proposal [38]; however, we applied it to all RGB channels instead of only green channel for the sake of comparison fairness. It stands for the state-of-the-art preprocessing method with identification accuracy of 90.21%. However, as clearly shown in Table 4 that it is improved by 2.41% with the proposed residual prediction model, this is a strong evidence of how multiscale features boost identification performance.

### 4.4. Experiment 3: Comparison of Identificaiton Network Structures

In order to verify the effectiveness of the proposed modified VGG network, we compared the identification accuracy while fixing the patch selection and residual prediction module. Model-level experiment results are shown in Table 5.

**Figure 7:**
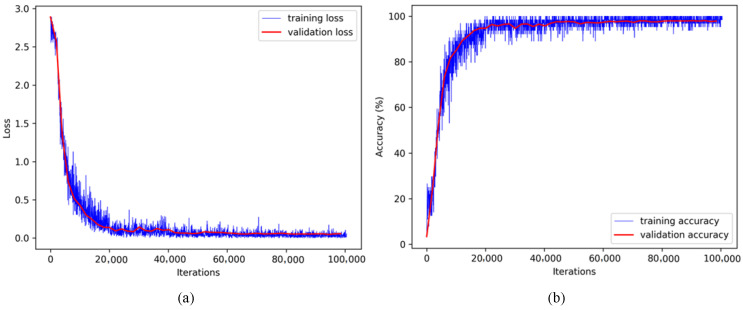
Convergence curves of the proposed modified VGG network. (**a**) Loss vs. iterations and (**b**) accuracy vs. iterations.

We also present the training history of the proposed method in Figure 7, where the loss and identification accuracy are plotted with respect to the number of iterations. It can be clearly seen that the proposed modified VGG network converges quickly (at around 20–30 epochs), where the loss stabilized at about 0.1. Moreover, there is no significant gap between the training and validation accuracy, indicating no overfitting tendency of the network.

### 4.5. Experiment 4: Comparison with State-of-the-Art-Methods

After we have discussed the effectiveness of the three fundamental blocks of the proposed method separately, we now evaluate its performance with other state-of-the-art methods at brand, model, and instance levels.

There have been many successful camera identification methods, most of which are based on convolutional neural networks. One may notice that, except in [9] where all patches are used for training, the number of training patches is usually smaller as compared to the proposed method. To compensate this shortage of training patches, the proposed multiple-criteria-based patch selection is adopted to replace those in [7,8,21,27] and [38]. Experiments are strictly conducted according to the data sets and evaluation protocol as discussed in Section 3.4 and Section 4.1. Identification accuracy results as well as training time are summarized in Table 6, obtained either by source code provided by authors [7], reimplementation of the network structure in their original papers [8,9,21,27], or with minor modification caused by patch size inconsistence [38].

The pioneering work [7] serves as a benchmark for our discussion. Note that the model-level accuracy of 78.86% is much lower as compared with that reported in the original paper (93%). This is due to different evaluation settings, suggesting that the proposed evaluation protocol is more critical. Meanwhile, it can be clearly observed that with the increasing difficulty in distinguishing different brands, models, and instances, identification accuracy drops sharply from 81.2% and 78.86% (brand-level accuracy and model-level accuracy, respectively) to 33.83% (instance-level accuracy). It is not surprising since shared common features also show a decreasing trend for these three tasks. Furthermore, the downsample operation in pooling layer is responsible for the poor performance in the instance level, which is commonly reported in CNN-based methods.

By comparison, identification accuracy improvement in the work of Tuama et al. [8] is obvious; a fixed 5×5 high-pass filter was imposed onto the input image to obtain residual-like images. Meanwhile, with similar CNN structures, training cost is also comparative with [7]. Initially designed for a smaller patch size of 36×36, the network in [21] is relatively simple, resulting in the shortest training time. However, it is only slightly inferior to [8] at the model and instance levels, with a surprising good brand-level identification rate (93.26%). This might be caused by the simple network structure in which only large-scale common features are better revealed.

In [38], the preprocessing is accomplished by the proposed augmented convolutional feature maps (ACFM), consisting of a nonlinear median filter residual and a constrained convolutional layer applied to the green channel in parallel. The network is originally designed for 256×256 patches with deeper network structure. In our reimplementation, minor modifications are applied to adapt to the 64×64 patch size setting: stride of conv2 layer is reduced from 2 to 1, while padding parameters are increased by 1 for conv2, conv3, and conv4 layers. One can observe obvious performance improvement at all brand, model, and instance levels, while training times is increased almost five times as compared to Bondi’s work [7].

Yang et al. employed another strategy [9] that no patch selection is involved. According to image contents, all patches are divided into three subsets: saturation, smoothness, and others, while three fusion residual networks are trained correspondingly to handle them. Network complexity is further increased by three parallel branches within each fusion residual network, leading to a training time of 46 hours that stands out at the top of all methods in comparison. Guided by the divide and conquer principle, it is not surprising that prominent performance improvement is obtained due to these efforts.

A dedicated designed remnant block was recently proposed in [27] for forensic feature-enriched residual learning at the camera model level. The original patch input is connected to all three cascaded remnant blocks by skip connections to avoid possible information loss. As shown in Table 6, high model level accuracy of 91.79% is reported at 365,000 iterations in our simulation, while better results can be expected through some structure adjustments for brand and instance levels. Note that the output feature map of each remnant block remains the same as the patch input (64×64), which may explain the relative long training time.

However, we can clearly see that the proposed method performs consistently best among all methods at all levels. Meanwhile, the computation complexity is limited, which is comparable to Bayar’s work [38]. The proposed multiple-criteria-based patch selection scheme plays an important role, as only 128 representative patches are selected as compared to more than 2000 image patches for each image. Meanwhile, the multiscale information is explored by granular level features with the proposed residual prediction model, which is more economic and flexible as compared with the content-based fusion network in [9]. With the modified VGG network, it is safe to draw the conclusion that the proposed method is more preferable in practical SCI applications.

### 4.6. Experiment5: Confusion Matrix Analysis

**Figure 8:**
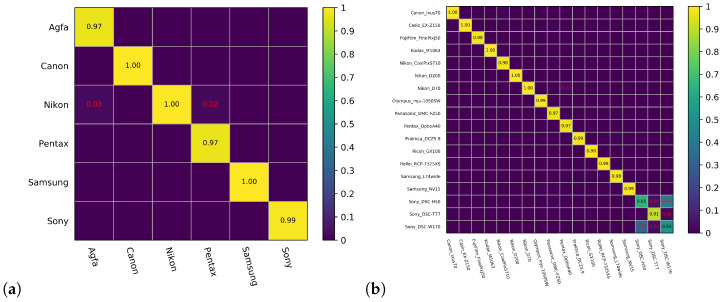
Confusion matrix of (**a**) brand level and (**b**) model level identification.

To gain further understanding of the identification performance on specific categories, we present confusion matrix analysis of the proposed method at three levels in detail. It can be clearly seen in the brand-level confusion matrix in Figure 8a that almost all six brands can reach to nearly 100% identification. However, some of the images taken by the Agfa and Pentax brand cameras are erroneously identified as images taken by the Nikon brand camera, indicating the built-in image processing algorithms by Nikon cameras share certain common features with these two brands.

From the classification confusion matrix visualization of the 18 camera models in Figure 8b, one can see that the classification accuracy of most camera models is higher than 97%. However, accuracy of the three Sony categories is significantly lower, as they greatly interfere with each other, which seriously affects the overall accuracy. This phenomenon has been reported in many papers [7,24]. One possible reason is that the hardware and software configurations of these camera models are similar during the production process, which makes their model features difficult to distinguish. Meanwhile, an insufficient number of training images may exacerbate this phenomenon. It is revealed in Table 1 that there are least images from models of Sony_DSC-H50 and Sony_DSC-W170, corresponding to the worst two identification rates. The number of images of Sony_DSC-T77 is moderate, while its result is slightly better than those of the other two models.

**Figure 9:**
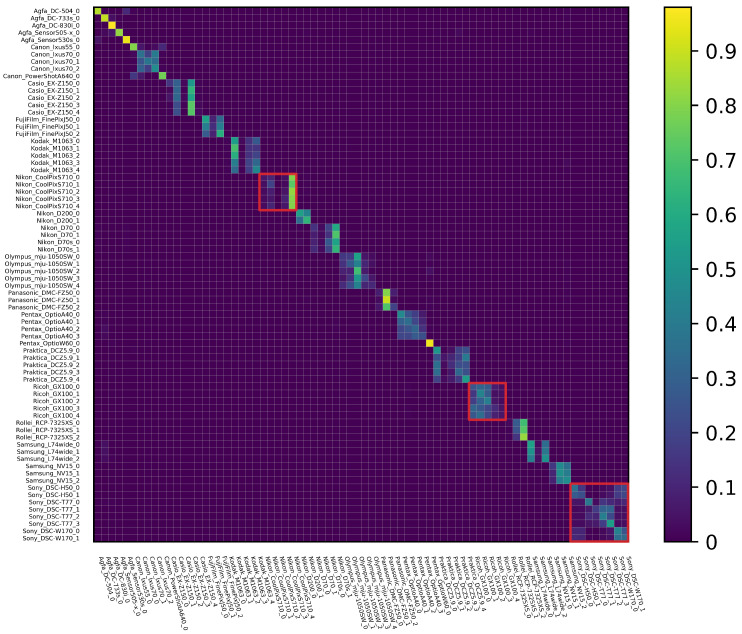
Confusion matrix of instance level identification.

The classification confusion matrix of the instance level identification is shown in Figure 9. As discussed before, instance-level identification is more difficult, especially for a 74-category classification problem. There is an obvious block effect shown in Figure 9; interference between camera instances of the same model is severe (see the Nikon_CoolPixS710, Ricoh_GX100, and Sony cases highlighted in red squares, for example), echoing the low identification accuracy of instance-level SCI (41.54%, as shown in Table 6). The topic of how to design deep structures that can efficiently exploit instance level features is a fruitful direction for future SCI studies.

### 4.7. Image Tampering Detection

Image tampering detection is a hot topic in the image forensic field; many algorithms have been proposed as potential solutions. As a composited image usually contains contents from different camera sources, SCI methods can be easily applied in the image tampering detection task. The smaller the patch size that SCI algorithms can stably work with, the better the tamper detection performance one can expect.

**Figure 10:**
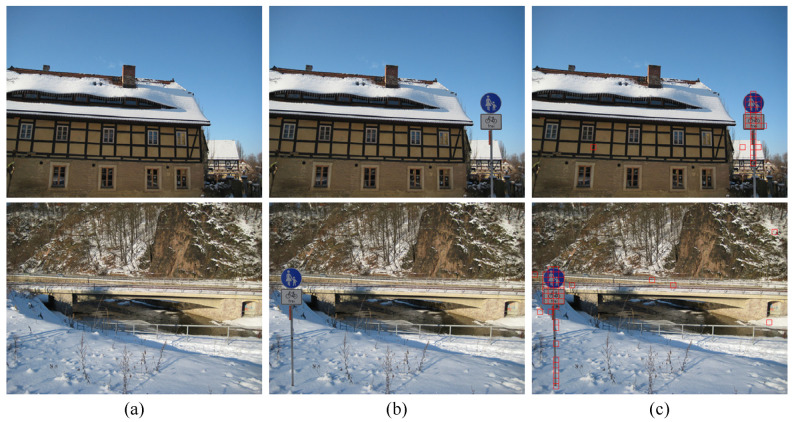
Image tampering detection. (**a**) Original images. (**b**) Tampered images. (**c**) Detection results.

An illustrative example is presented in Figure 10, where image parts of traffic signs from Kodak_M1063 are carefully spliced into two Canon_Ixus70 images. All 64×64 nonoverlapping patches in the tampered images (Figure 10b) are identified according to the trained model-level network with the proposed method. Taking the majority voting results of all patches as the model ID of the whole image, misclassified patches are marked with red squares, considered as the tamper detection results shown in Figure 10c.

It can be clearly observed that most of the tampered contents can be correctly identified. Note that the discontinuities and false positives shown in Figure 10c can be easily removed by imposing spatial connectivity and consistency assumptions with the detection results. This is because with the fast development of cameras, image content smaller than 64×64 is meaningless. Isolated individual detected patches could be eliminated, while separated regions should be merged together. Logical and morphological operations can also be involved in subsequent image tampering detection algorithms, which is one of the future directions to be studied.

### 4.8. Failure Cases Analysis

By comparison results conducted on the proposed evaluation protocol, we see that the proposed method outperforms several state-of-the-art SCI algorithms. However, it should be noted that there are still some limitations that it may fail in some situations. Analysis of failure cases helps to reveal more on shortcomings of the algorithm and problems to be solved.

**Figure 11:**
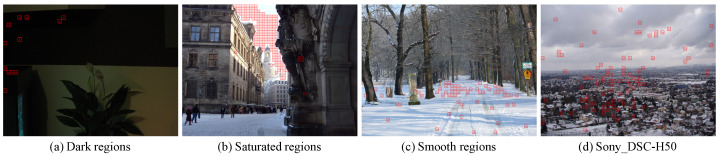
Failure examples of the proposed method at model level identification.

Hereby, we discuss these limitations by some failure cases at the model level identification shown in Figure 11. Although most patches are correctly identified, some patches in dark regions of Figure 11a and a considerable number of saturated sky patches in Figure 11b are misclassified. It was reported in [4] that the instance level camera fingerprint photoresponse nonuniformity noise (PRNU) term is not present in saturated regions. Similarly, little evidence of model level feature is observed in such regions. Meanwhile, identification of a certain number of patches in smooth regions failed, as shown in Figure 11c. This is probably due to the fact that smooth regions are easier to process as compared to edge and texture regions. Consequently, processing algorithms employed by different camera models are not as discriminative in such regions. To address these problems, special strategies for dark, saturated, and smooth regions should be considered. Moreover, as can be clearly seen in Figure 11d, misclassification among the three Sony categories is quite severe. Although it is commonly reported in many literatures [7,9,24], underlying reasons needs to be further revealed.

## 5. Conclusions

In this paper, we developed an efficient source camera identification approach, consisting of three fundamental blocks of multiple-criteria-based patch selection, fine-grained multiscale residual prediction, and modified VGG identification. It performs well under the proposed patch level evaluation protocol at the brand, model, and instance levels, in terms of both identification accuracy and computation efficiency. Applications in image tampering detection and failure cases analysis are also presented. The experimental results reveal that identification of dark and saturated regions and instance-level identification are important problems to be studied in the future.

## Figures and Tables

**Table 1:** Details of camera models used in experiments.

No.	Camera Model	Resolution	No. Images
0	Canon_Ixus70	3072×2304	363
1	Casio_EX-Z150	3264×2448	692
2	FujiFilm_FinePixJ50	3264×2448	385
3	Kodak_M1063	3664×2748	1698
4	Nikon_CoolPixS710	4352×3264	695
5	Nikon_D200	3872×2592	373
6	Nikon_D70	3008×2000	373
7	Olympus_mju-1050SW	3648×2736	782
8	Panasonic_DMC-FZ50	3648×2736	564
9	Pentax_OptioA40	4000×3000	405
10	Praktica_DCZ5.9	2560×1920	766
11	Ricoh_GX100	3648×2736	559
12	Rollei_RCP-7325XS	3072×2304	377
13	Samsung_L74wide	3072×2304	441
14	Samsung_NV15	3648×2736	412
15	Sony_DSC-H50	3456×2592	253
16	Sony_DSC-T77	3648×2736	492
17	Sony_DSC-W170	3648×2736	201

**Table 2:** Comparison of model level identification accuracy with varying number of training patches per image.

No. of Patches	32	64	128	256
Accuracy (100%)	85.90	88.69	91.70	90.81

**Table 3:** Comparison of model level identification accuracy with varying parameter settings.

*T*	128	32	32	32	64	64	64
*k*	0	16	32	96	16	32	64
*n*	0	6	3	1	4	2	1
Accuracy (100%)	84.16	86.15	86.22	86.76	87.37	86.39	86.65

**Table 4:** Comparison of model level identification accuracy of different preprocessing methods.

Method	Accuracy (%)
None	87.37
Fixed high-pass filter [8]	88.79
Mean filter	89.84
Constrained convolutional layer [38]	90.21
Proposed	92.62

**Table 5:** Comparison of model-level identification accuracy of different identification networks.

Method	Accuracy (%)
Bondi Network [7]	90.38
Residual network (5 × 5) [9]	90.93
Content adaptive fusion residual networks [9]	91.90
Hierarchical Multitask Learning [10]	92.18
Modified VGG network (Proposed)	92.62

**Table 6:** SCI accuracy comparison with state-of-the-art methods at three levels.

Method	Brand (%)	Model (%)	Instance (%)	Training Time
Bondi [7]	81.20	78.86	33.83	0.67 h
Tuama [8]	89.19	83.90	31.36	0.68 h
Huang [21]	93.26	82.14	31.01	0.52 h
Bayar [38]	93.21	87.31	35.53	3.23 h
Yang [9]	97.74	88.73	40.26	46 h
Rafi [27]	96.96	91.79	35.31	8.58 h
Proposed	98.14	92.62	41.54	3.95 h
